# Relação entre o Índice Imune-inflamação Sistêmico e Circulação Colateral Coronariana em Pacientes com Oclusão Total Crônica

**DOI:** 10.36660/abc.20210414

**Published:** 2022-06-06

**Authors:** Mehmet Koray Adali, Ipek Buber, Gursel Sen, Samet Yilmaz

**Affiliations:** 1 Pamukkale University Faculty of Medicine Departamento de Cardiologia Denizli Turquia Pamukkale University - Faculty of Medicine, Departamento de Cardiologia, Denizli – Turquia

**Keywords:** Circulação Colateral, Oclusão Coronária, Vasos Coronários

## Abstract

**Fundamento:**

A inflamação desempenha um papel fundamental no início e na progressão da doença arterial coronariana (DAC). O Índice Imune-inflamação Sistêmico (SII) é um novo parâmetro inflamatório que demonstrou estar associado à DAC.

**Objetivos:**

Este estudo teve como objetivo investigar a relação entre o SII e a circulação colateral coronariana (CCC) em pacientes com DAC estável e oclusão crônica total (OTC).

**Métodos:**

Os pacientes foram divididos em dois grupos, com CCC deficiente e CCC boa, de acordo com a Classificação Rentrop. Noventa e quatro pacientes apresentavam CCC deficiente e 81 pacientes CCC boa. Os parâmetros de inflamação foram calculados a partir dos resultados laboratoriais. O nível de significância estatística aplicado foi de 0,05.

**Resultados:**

Alto nível de SII (OR: 1,003, IC 95%: 1,001-1,004, p<0,001), ausência de OTC na ACD (artéria coronária direita) (OR: 0,204, IC 95%: 0,096-0,436, p<0,001) e baixo escore de Gensini (OR: 0,980, IC 95%: 0,962-0,998, p=0,028) foram significantemente associados com CCC deficiente. O valor de corte do SII foi de 679,96 para o maior poder preditivo de CCC deficiente, com sensibilidade de 74,5% e especificidade de 43,2%. As taxas de mortalidade foram semelhantes entre os dois grupos durante um seguimento médio de 21,5±10,8 meses (p=0,107).

**Conclusões:**

Alto nível de SII, ausência de OTC na artéria coronária direita e baixo escore de Gensini foram significantemente relacionados à CCC deficiente. O uso rápido e custo-efetivo de novos marcadores inflamatórios na prática clínica orienta o prognóstico da DAC.

## Introdução

A oclusão total crônica (OTC) é uma forma de doença arterial coronariana (DAC) que se caracteriza pela oclusão completa ou quase completa das artérias coronárias epicárdicas por pelo menos 3 meses e apresenta pior evolução clínica. A OTC tem incidência que varia de 18% a 52% a partir do exame de cineangiocoronariografia.^1^ A circulação colateral coronariana (CCC) é uma resposta adaptativa que se desenvolve para manter a perfusão do tecido miocárdico em pacientes com lesões coronarianas estenóticas ou oclusivas. Em uma meta-análise de Meier et al., foi relatado que pacientes com boa CCC apresentaram 36% menos mortalidade do que pacientes com CCC deficiente.^2^

O grau de estenose coronariana, a presença de diabetes mellitus, a prática de exercícios físicos, as crises anginosas, os mediadores que afetam a angiogênese, como o fator de crescimento endotelial vascular (VEGF, do inglês *vascular endothelial growth factor* ), e os níveis de células inflamatórias afetam o desenvolvimento colateral coronariano.^2 - 6^ Por causa dos processos inflamatórios que afetam a DAC em larga escala, os parâmetros inflamatórios obtidos de exames de rotina, como hemograma completo (CBC, do inglês *complete blood count* ) e bioquímica sanguínea, são frequentemente utilizados em uma ampla variedade de estudos clínicos como preditores tanto do desenvolvimento colateral coronariano quanto da gravidade da DAC.^7 - 9^

O Índice Imune-inflamação Sistêmico (SII, do inglês *Systemic immune-inflammation index* ), um novo parâmetro inflamatório, foi considerado um preditor independente de eventos cardiovasculares em pacientes com DAC submetidos à intervenção coronária percutânea (ICP).^10^ Embora muitos parâmetros inflamatórios tenham sido estudados em pacientes com DAC com OTC, a razão de monócitos para lipoproteína de alta densidade (MHR, do inglês *monocyte to high-density lipoprotein ratio* ) e o SII não foram anteriormente estudados na literatura nesta situação clínica. Portanto, nosso objetivo foi investigar o valor preditor do SII no desenvolvimento colateral coronariano em pacientes com DAC estável com OTC.

## Métodos

### População e desenho do estudo

**Figura 1:**
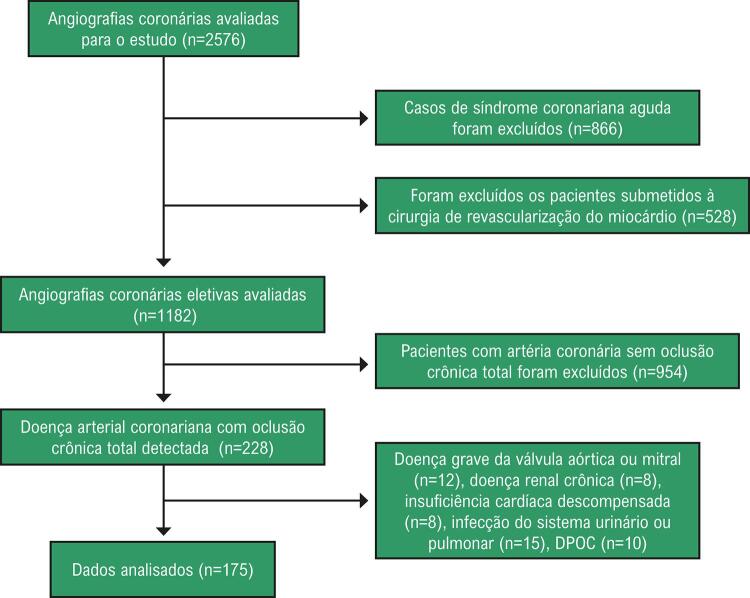
Fluxograma de inclusão de pacientes.

Após a aprovação do comitê de ética local, 2.576 procedimentos de cineangiocoronariografia foram avaliados entre janeiro de 2018 e julho de 2020 a partir dos registros do instituto. O fluxograma de inclusão dos pacientes pode ser visto na Figura 1 . Cento e setenta e cinco pacientes com DAC estável com OTC foram incluídos no estudo e foram agrupados de acordo com a classificação de Rentrop^11^ quanto ao desenvolvimento colateral coronariano na OTC. Os pacientes foram divididos em dois grupos com CCC deficiente (Grau 0 e 1) e CCC boa (Grau 2 e 3). Noventa e quatro pacientes apresentavam CCC deficiente e 81 pacientes tinham CCC boa. As características clínicas e demográficas, fatores de risco para DAC, medicamentos, resultados laboratoriais, eletrocardiograma (ECG) e registros de mortalidade dos pacientes foram registrados no banco de dados do hospital. SII, MHR, razão plaquetas/linfócitos (PLR, do inglês *platelet to lymphocyte ratio* ) e razão neutrófilos-linfócitos (NLR, do inglês *neutrophil-lymphocyte ratio* ) foram calculados a partir dos resultados laboratoriais de CBC e parâmetros bioquímicos. O valor do SII foi calculado utilizando a fórmula SII = (P × N) / L. Na fórmula P, N e L simbolizam plaquetas, neutrófilos e linfócitos, respectivamente. A hipertensão foi definida como registro prévio de pressão arterial sistólica de 140 mm Hg e/ou pressão arterial diastólica de 90 mm Hg em pelo menos duas medições ou uso ativo de qualquer agente anti-hipertensivo. Diabetes mellitus foi definida como um nível plasmático de glicose em jejum acima de 126 mg/dL, um nível de glicose acima de 200 mg/dL ou um nível de hemoglobina glicada acima de 6,5% em qualquer medida, ou o uso ativo de um agente antidiabético. Colesterol total >200 mg/dL e triglicérides >150 mg/dL ou o uso ativo de medicamentos anti-hiperlipidêmicos foram considerados como hiperlipidemia.

Foram excluídos do estudo pacientes com patologia valvar cardíaca moderada a grave, síndrome coronariana aguda nos últimos 3 meses, insuficiência cardíaca descompensada (classe III ou IV do NYHA), doença pulmonar obstrutiva crônica, sinais clínicos de infecção ativa, insuficiência renal aguda ou crônica, insuficiência hepática e aqueles com histórico de malignidade, cirurgia de revascularização do miocárdio (CRM), embolia pulmonar, doença inflamatória crônica ou autoimune e aqueles submetidos a transplante renal-hepático.

O estudo está em conformidade com os princípios descritos na Declaração de Helsinque.

### Avaliação da circulação colateral coronariana

A cineangiocoronariografia foi indicada para pacientes com dor torácica ou aqueles submetidos a testes não-invasivos que mostraram isquemia miocárdica. A cineangiocoronariografia foi realizada por acesso transfemoral ou transradial utilizando a técnica de rotina de Judkins. A OTC foi definida como uma oclusão total de uma artéria coronária com fluxo distal TIMI 0 há pelo menos 3 meses. Foram incluídos no estudo pacientes que apresentavam pelo menos uma artéria coronária com OTC. A CCC foi avaliada por dois cardiologistas com mascaramento. A CCC foi avaliada utilizando o sistema de escore desenvolvido por Cohen et al. (classificação Rentrop).^11^ De acordo com o sistema de classificação: Grau 0, sem enchimento visível de qualquer coronária colateral; Grau 1, enchimento de ramos laterais da artéria a ser dilatada por canais colaterais sem visualização da parte epicárdica; Grau 2, enchimento parcial da parte epicárdica por canais colaterais; Grau 3, enchimento completo da artéria epicárdica sendo dilatada através de canais colaterais.

### Análise estatística

Todos os dados foram analisados utilizando o pacote estatístico SPSS 22.0 (SPSS Inc., Chicago, IL, EUA). As variáveis contínuas foram relatadas como média ± desvio padrão e as variáveis categóricas como frequências absolutas e relativas. O teste de Kolmogorov-Smirnov foi utilizado para determinar a normalidade dos dados. O teste *t* de Student Independente foi usado para comparar variáveis com distribuição normal. As variáveis categóricas foram comparadas com o teste do χ^2^ ou teste exato de Fisher. Um valor de p <0,05 foi considerado estatisticamente significante. Os efeitos de diferentes variáveis na CCC deficiente foram avaliados por análise de regressão logística *backward* . A inclusão de covariáveis no modelo multivariável foi determinada pela seleção daquelas que exibiram um valor de p bilateral < 0,10 em análises não ajustadas. A inclusão de covariáveis adicionais foi determinada através de um processo de seleção *stepwise-backward* até que todas as outras variáveis do modelo exibissem p < 0,10. A análise da curva ROC ( *Receiver Operating Characteristic* ) foi utilizada para determinar o melhor valor de corte do nível de SII na previsão de CCC deficiente.

## Resultados

No total, 175 pacientes com DAC estável com OTC foram incluídos no estudo. A média de idade dos pacientes foi de 68,2±10,9 e 80,6% dos pacientes eram do sexo masculino. Havia dois grupos: um com 94 pacientes no grupo com CCC deficiente (Grau de Rentrop 0 ou 1) e 81 pacientes no grupo com CCC boa (Grau de Rentrop 2 ou 3). Idade, gênero, presença de hipertensão, diabetes, hiperlipidemia, histórico familiar de doença cardiovascular (DCV), IM anterior e medicações foram semelhantes entre os dois grupos. Em todos os pacientes, a localização da OTC foi maior na artéria coronária direita (ACD) e estatisticamente mais alta no grupo com CCC boa. A taxa de doença multiarterial (≥ 2 DAC) foi discreta e o escore de Gensini foi significativamente maior no grupo com CCC boa. As taxas de mortalidade foram semelhantes entre os dois grupos durante um seguimento médio de 21,5±10,8 meses. As características basais demográficas, clínicas, fatores de risco para DAC e medicação prévia dos pacientes são mostradas na Tabela 1 .

**Tabela 1:** Características demográficas e clínicas basais da população estudada

Características	Todos os pacientes (n = 175)	Circulação colateral coronariana		p-valor
		Deficiente (n = 94)	Boa (n = 81)	
Idade (anos), média±DP	68,2±10,9	69,1±11,2	67,3±10,5	0,275
Masculino, n (%)	141 (80,6)	74 (78,7)	67 (82,7)	0,568
PAS, mm Hg	138,4±20,44	127,8±16	129,7±18,6	0,478
PAD, mm Hg	74,19±12,79	76,1±11,6	76,7±13	0,742
Fumante atual, n (%)	36 (20,6)	18 (19,1)	18 (22,2)	0,708
Hipertensão, n (%)	103 (58,9)	59 (62,8)	44 (54,3)	0,283
Diabetes mellitus, n (%)	69 (39,4)	38 (40,4)	31 (38,2)	0,877
Hiperlipidemia, n (%)	15 (8,6)	10 (10,6)	5 (6,1)	0,418
História familiar de DCV, n (%)	15 (8,6)	10 (10,6)	5 (6,1)	0,418
MI anterior, n (%)	77 (44)	44 (46,8)	37 (45,7)	0,448
**Medicação, n (%)**				
ASA	92 (52,6)	56 (59,6)	36 (44,4)	0,050
Inibidor de P2Y12	42 (24)	27 (28,7)	15 (18,5)	0,155
Estatina	52 (29,7)	29 (30,8)	23 (28,4)	0,743
IECA/BRA	71 (40,6)	40 (42,5)	31 (38,2)	0,644
Betabloqueador	83 (47,4)	48 (51)	35 (43,2)	0,363
Bloqueador de canais de cálcio	31 (17,7)	18 (19,1)	13 (16)	0,692
FE, %, média±DP	47,1±12,2	46,1±12,2	48,3±12,2	0,224
Doença multiarterial, n (%)	121 (69,1)	59 (62,8)	62 (76,5)	0,071
**Localização do OTC, n (%)**				
ADA	46 (26,3)	30 (31,9)	16 (19,7)	0,085
Cx	25 (14,3)	16 (17)	9 (11,1)	0,287
ACD	96 (54,9)	38 (40,4)	58 (71,6)	<0,001
Outros	22 (12,6)	13 (13,8)	9 (11,1)	0,652
Escore de Gensini, média±DP	58,6±23,2	55±18,6	62,8±27,1	0,025
Mortalidade, n (%)	41 (23,4)	27 (28,7)	14 (17,3)	0,107

*IECA: inibidor da enzima conversora de angiotensina; BRA: bloqueador do receptor de angiotensina; ASA: ácido acetilsalicílico; OTC: oclusão crônica total; CVD: doença cardiovascular; Cx: artéria coronária circunflexa; PAD: pressão arterial diastólica; FE: fração de ejeção; ADA: artéria coronária descendente anterior esquerda; IM: infarto do miocárdio; ACD: artéria coronária direita; PAS: pressão arterial sistólica.*

Os resultados laboratoriais e os parâmetros inflamatórios de ambos os grupos são mostrados na Tabela 2 . A contagem de plaquetas, leucócitos e neutrófilos foi notavelmente maior no grupo com CCC deficiente. A contagem de linfócitos foi maior no grupo com CCC boa. Hemoglobina, contagem de monócitos, taxa de filtração glomerular e níveis de colesterol foram semelhantes entre os dois grupos. Entre os parâmetros inflamatórios, a proteína C-reativa (PCR) e a MHR não apresentaram diferenças significantes entre os grupos, mas os valores de NLR, PLR e SII foram estatisticamente menores no grupo com CCC boa.

**Tabela 2:** Resultados laboratoriais e parâmetros inflamatórios dos pacientes

Características	Todos os pacientes (n = 175)	Circulação colateral coronariana		p-valor
		Deficiente (n = 94)	Boa (n = 81)	
**Resultados laboratoriais, média±DP**				
Hemoglobina, g/L	13,2±2	13±2,2	13,5±1,8	0,139
Contagem de plaquetas, 103/µL	253,8±60,4	267,5±65,2	237,9±50,1	0,001
Contagem de leucócitos, 103/µL	9,4±2,8	10,1±3,1	8,7±2	0,001
Contagem de neutrófilos, 103/µL	6,5±2,5	7,3±2,9	5,6±1,6	<0,001
Contagem de linfócitos, 103/µL	2,1±0,9	1,9±0,85	2,2±0,88	0,028
Contagem de monócitos, 103/µL	0,62±0,25	0,62±0,26	0,62±0,25	0,251
Creatinina, mg/dL	1,04±0,28	1,04±0,27	1,04±0,29	0,895
TFG, mL/min	74,1±20,1	73,2±19,9	75,1±20,5	0,526
Colesterol total, mg/dL	180,9±46,3	126±70,3	113±46,3	0,583
HDL-C, mg/dL	40,8±12,3	42±11,4	39,5±13,2	0,174
LDL-C, mg/dL	107,4±42,8	104,3±43,2	110,8±42,3	0,319
Triglicérides, mg/dL	171±112	167,9±99,8	174,6±125,2	0,694
PCR, mg/L	13,2±22	14,2±23,4	12,1±20,2	0,533
MHR	17,1±10,1	15,9±8	18,4±12	0,112
NLR	4,1±3,7	5,1±4,7	2,9±1,3	<0,001
PLR	152,5±108,7	179,5±136,7	121,2±46	<0,001
SII	1030,6±1008,9	1335,3±1275,4	679,9±295,3	<0,001

*PCR: proteína C reativa; TFG: taxa de filtração glomerular; HDL-C: colesterol de lipoproteína de alta densidade; LDL-C: colesterol de lipoproteína de baixa densidade; MHR: razão de monócitos para lipoproteína de alta densidade; NLR: razão neutrófilos/linfócitos; PLR: razão plaquetas/linfócitos; SII: Índice Imune-inflamação Sistêmico.*

Foi realizada uma análise multivariada de regressão *backward* dos fatores de risco para CCC deficiente. O modelo incluiu idade, gênero, hipertensão, diabetes, hiperlipidemia, tabagismo atual, IM anterior, doença multiarterial, frequência cardíaca, fração de ejeção, uso de ácido acetilsalicílico, uso de estatina, presença de OTC na ACD, estado colateral, escore de Gensini, NLR, PLR e SII. A análise mostrou que a ausência de OTC na ACD e o baixo escore de Gensini estavam relacionados à CCC deficiente. Além disso, um nível alto de SII foi significantemente associado a uma CCC deficiente ( Tabela 3 ).

**Tabela 3:** Análise multivariada de regressão backward dos fatores de risco para CCC deficiente

Variáveis *,**,***	OR, IC 95%	p-valor
Hiperlipidemia	0,313 (0,091-1,071)	0,064
OTC na ACD	0,204 (0,096-0,436)	<0,001
Escore de Gensini	0,980 (0,962-0,998)	0,028
Uso de ASA	0,526 (0,249-1,111)	0,092
SII	1,003 (1,001-1,004)	<0,001

ASA: ácido acetilsalicílico; CCC: circulação colateral coronariana; IC: intervalo de confiança; ACD: artéria coronária direita; OR: odds ratio; SII: Índice Imune-inflamação Sistêmico. * R^2^ de Nagelkerke: 0,432. **O modelo incluiu idade, gênero, hipertensão, diabetes, hiperlipidemia, tabagismo atual, IM anterior, doença multiarterial, frequência cardíaca, fração de ejeção, uso de ácido acetilsalicílico, uso de estatina, presença de obstrução crônica total na artéria coronária direita, grau de colateral, Escore de Gensini, NLR, PLR e, SII. ***A seleção de covariáveis para modelos multivariáveis é explicada na seção Métodos. Salvo indicação em contrário, odds ratio são interpretados como a presença (vs. ausência) de cada variável categórica ou um aumento de 1 unidade de cada variável contínua.

**Figura 2:**
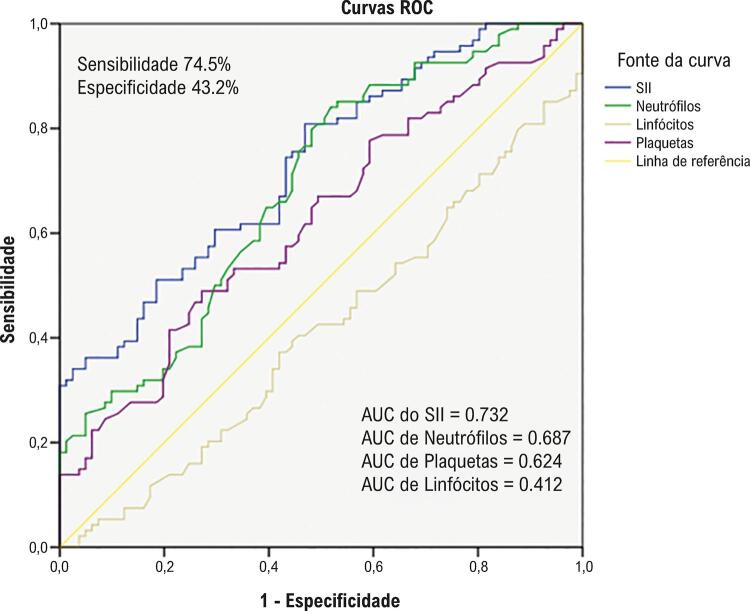
Curvas ROC de pacientes com CCC deficiente predita pelo SII.

AUC: Área sob a curva; CCC: circulação colateral coronariana; ROC: Característica de Operação do Receptor; SII: Índice Imune-inflamação Sistêmico.

Avaliamos o valor preditor do SII para CCC deficiente em uma análise da curva ROC. Quando o valor de corte do SII foi estabelecido em 679,96, o poder preditivo de CCC deficiente foi o mais alto, com sensibilidade de 74,5% e especificidade de 43,2% (AUC: 0,732; IC 95%, 0,659–0,804, p< 0,001) ( Figura 2 ).

## Discussão

Que seja de nosso conhecimento, este é o primeiro estudo que avalia a relação entre SII e CCC em pacientes com DAC estável e OTC. No presente estudo, verificamos que um alto nível de SII, ausência de OTC na ACD, e baixo escore de Gensini estavam relacionados a uma CCC deficiente.

Os vasos colaterais coronários são um mecanismo adaptativo que é ativado por eventos isquêmicos miocárdicos crônicos ou recorrentes; eles progridem gradualmente e protegem da isquemia miocárdica e suas complicações associadas.^2 , 12^ Hipóxia, aumento do potencial redox ou estresse de cisalhamento e algumas expressões genômicas causam ativação de células endoteliais e o início das cascatas inflamatórias.^13^ Devido ao papel central da inflamação no início e na progressão da DAC, vários estudos têm sido realizados para identificar o efeito dos processos inflamatórios na CCC. Valores altos de PCR, NLR, PLR, relação PCR/albumina (CAR, do inglês *CRP to albumin ratio* ) e razão fibrinogênio/albumina (FAR, do inglês *fibrinogen to albumin ratio* ) têm sido utilizados para este propósito.^7 , 8 , 14 - 16^

Acar et al. descobriram que a PLR foi um preditor de fluxo colateral deficiente em pacientes com angina estável e OTC.^7^ Em outro estudo, a NLR foi associada à redução do fluxo colateral coronariano na DAC com OTC.^8^ Também encontramos níveis elevados de PLR e NLR no grupo com CCC deficiente (p<0,001), mas essa significância não foi encontrada na análise de regressão.

O aumento do nível de MHR foi identificado como um preditor de alto escore SYNTAX em pacientes com DAC estável.^9^ No presente estudo, também objetivamos investigar o efeito desse parâmetro inflamatório no desenvolvimento da CCC, mas não houve diferença significante em termos de MHR.

O SII foi desenvolvido a partir de células inflamatórias, incluindo contagem de plaquetas, neutrófilos e linfócitos. Em primeiro lugar, tem sido associado a mau prognóstico em muitos tipos de câncer.^17 , 18^ Utilizando a coorte de Dongfeng-Tongji, Xu et al. descobriram que o SII estava associado a trombocitose, inflamação e desenvolvimento de doença cerebrovascular em 13.929 adultos de meia-idade e idosos sem DCV e câncer, em um seguimento médio de 8,28 anos.^19^ Yang et al. demonstraram que o nível elevado de SII está independentemente associado ao aumento do risco de morte cardiovascular, IAM não-fatal, acidente vascular cerebral não-fatal e hospitalização por insuficiência cardíaca em 5.206 pacientes com DAC submetidos a ICP.^10^ Neste estudo, um ponto de corte ideal de SII (≥694,3) foi identificado para eventos cardiovasculares adversos maiores (ECAM) na coorte com DAC. Da mesma forma, em nosso estudo, encontramos um ponto de corte ideal do SII de 679,96 para a melhor predição de CCC deficiente, com sensibilidade de 74,5% e especificidade de 43,2%.

O efeito da CCC na mortalidade é discutível. Em uma meta-análise que incluiu mais de 3.000 pacientes, Allahwala et al. indicaram que uma CCC robusta não está associada a menores taxas de infarto agudo do miocárdio ou mortalidade por todas as causas, mas aumenta a chance de sucesso da ICP.^1^ Por outro lado, Meier et al. demonstraram que a alta colateralização teve efeito protetor e reduziu em 36% o risco de mortalidade em comparação com pacientes com baixa colateralização.^2^ Entretanto, em nosso estudo, não houve diferença significante nas taxas de mortalidade durante 21,5±10,8 meses de seguimento.

Este estudo tem algumas limitações. Primeiro, havia um número bastante pequeno de pacientes e o estudo foi transversal, unicêntrico, com desenho retrospectivo. Sendo assim, a população amostral selecionada pode não refletir toda a coorte e, portanto, mais estudos são necessários. Em segundo lugar, todas as medidas e parâmetros laboratoriais foram avaliados apenas uma vez durante o seguimento. Finalmente, expressões gênicas específicas, parâmetros inflamatórios como VEGF e TNF-α não foram medidos, de modo que essas medidas podem servir como suporte para demonstrar uma associação da CCC deficiente com o SII.

## Conclusão

Neste estudo, verificamos que o alto nível de SII, ausência de OTC na ACD e baixo escore de Gensini foram significativamente relacionados à CCC deficiente. É importante determinar rapidamente o estado inflamatório a partir dos resultados laboratoriais de sangue e para determinar a CCC deficiente e os pacientes de alto risco que resultam em alta mortalidade de pacientes com DAC. O SII é um parâmetro inflamatório fácil de calcular a partir do hemograma completo e pode ser muito útil para identificar pacientes de alto risco com CCC deficiente.

## References

[1] Allahwala UK, Nour D, Bhatia K, Ward MR, Lo S, Weaver JC, et al. Prognostic Impact of Collaterals in Patients with a Coronary Chronic Total Occlusion: A Meta-analysis of Over 3,000 Patients. Catheter Cardiovasc Interv. 2021;97(6):771-7. doi: 10.1002/ccd.29348. 10.1002/ccd.29348 33118694

[2] Meier P, Hemingway H, Lansky AJ, Knapp G, Pitt B, Seiler C. The Impact of the Coronary Collateral Circulation on Mortality: A Meta-analysis. Eur Heart J. 2012;33(5):614-21. doi: 10.1093/eurheartj/ehr308. 10.1093/eurheartj/ehr308 21969521

[3] Werner GS, Ferrari M, Heinke S, Kuethe F, Surber R, Richartz BM, et al. Angiographic Assessment of Collateral Connections in Comparison with Invasively Determined Collateral Function in Chronic Coronary Occlusions. Circulation. 2003;107(15):1972-7. doi: 10.1161/01.CIR.0000061953.72662.3A. 10.1161/01.CIR.0000061953.72662.3A 12665484

[4] Shen Y, Ding FH, Dai Y, Wang XQ, Zhang RY, Lu L, et al. Reduced Coronary Collateralization in Type 2 Diabetic Patients with Chronic Total Occlusion. Cardiovasc Diabetol. 2018;17(1):26. doi: 10.1186/s12933-018-0671-6. 10.1186/s12933-018-0671-6 PMC5804044 29422093

[5] Dai Y, Chang S, Wang S, Shen Y, Li C, Huang Z, et al. The Preservation Effect of Coronary Collateral Circulation on Left Ventricular Function in Chronic Total Occlusion and its Association with the Expression of Vascular Endothelial Growth Factor A. Adv Clin Exp Med. 2020;29(4):493-7. doi: 10.17219/acem/104535. 10.17219/acem/104535 32338833

[6] Imhof BA, Aurrand-Lions M. Angiogenesis and Inflammation Face Off. Nat Med. 2006;12(2):171-2. doi: 10.1038/nm0206-171. 10.1038/nm0206-171 16462798

[7] Açar G, Kalkan ME, Avci A, Alizade E, Tabakci MM, Toprak C, et al. The Relation of Platelet-lymphocyte Ratio and Coronary Collateral Circulation in Patients with Stable Angina Pectoris and Chronic Total Occlusion. Clin Appl Thromb Hemost. 2015;21(5):462-8. doi: 10.1177/1076029613508599. 10.1177/1076029613508599 24142833

[8] Akın F, Ayça B, Çelik Ö, Şahin C. Predictors of Poor Coronary Collateral Development in Patients with Stable Coronary Artery Disease: Neutrophil-to-Lymphocyte Ratio and Platelets. Anatol J Cardiol. 2015;15(3):218-23. doi: 10.5152/akd.2014.5263. 10.5152/akd.2014.5263 PMC5337058 25880175

[9] Akboga MK, Balci KG, Maden O, Ertem AG, Kirbas O, Yayla C, et al. Usefulness of Monocyte to HDL-cholesterol Ratio to Predict High SYNTAX Score in Patients with Stable Coronary Artery Disease. Biomark Med. 2016;10(4):375-83. doi: 10.2217/bmm-2015-0050. 10.2217/bmm-2015-0050 26999570

[10] Yang YL, Wu CH, Hsu PF, Chen SC, Huang SS, Chan WL, et al. Systemic Immune-inflammation Index (SII) Predicted Clinical Outcome in Patients with Coronary Artery Disease. Eur J Clin Invest. 2020;50(5):e13230. doi: 10.1111/eci.13230. 10.1111/eci.13230 32291748

[11] Cohen M, Rentrop KP. Limitation of Myocardial Ischemia by Collateral Circulation During Sudden Controlled Coronary Artery Occlusion in Human Subjects: A Prospective Study. Circulation. 1986;74(3):469-76. doi: 10.1161/01.cir.74.3.469. 10.1161/01.cir.74.3.469 2943529

[12] Khand A, Fisher M, Jones J, Patel B, Perry R, Mitsudo K. The Collateral Circulation of the Heart in Coronary Total Arterial Occlusions in Man: Systematic Review of Assessment and Pathophysiology. Am Heart J. 2013;166(6):941-52. doi: 10.1016/j.ahj.2013.09.010. 10.1016/j.ahj.2013.09.010 24268207

[13] Allahwala UK, Khachigian LM, Nour D, Ridiandres A, Billah M, Ward M, et al. Recruitment and Maturation of the Coronary Collateral Circulation: Current Understanding and Perspectives in Arteriogenesis. Microvasc Res. 2020;132:104058. doi: 10.1016/j.mvr.2020.104058. 10.1016/j.mvr.2020.104058 32798552

[14] Kelesoglu S, Yilmaz Y, Elcık D. Relationship Between C-Reactive Protein to Albumin Ratio and Coronary Collateral Circulation in Patients with Stable Coronary Artery Disease. Angiology. 2021;72(9):829-35. doi: 10.1177/00033197211004392. 10.1177/00033197211004392 33759588

[15] Zhao Y, Wang S, Yang J, Lin Z, Chen Q. Association of Fibrinogen/Albumin Ratio and Coronary Collateral Circulation in Stable Coronary Artery Disease Patients. Biomark Med. 2020;14(16):1513-20. doi: 10.2217/bmm-2020-0333. 10.2217/bmm-2020-0333 33200965

[16] Gulec S, Ozdemir AO, Maradit-Kremers H, Dincer I, Atmaca Y, Erol C. Elevated Levels of C-Reactive Protein are Associated with Impaired Coronary Collateral Development. Eur J Clin Invest. 2006;36(6):369-75. doi: 10.1111/j.1365-2362.2006.01641.x. 10.1111/j.1365-2362.2006.01641.x 16684119

[17] Hu B, Yang XR, Xu Y, Sun YF, Sun C, Guo W, et al. Systemic Immune-Inflammation Index Predicts Prognosis of Patients After Curative Resection for Hepatocellular Carcinoma. Clin Cancer Res. 2014;20(23):6212-22. doi: 10.1158/1078-0432.CCR-14-0442. 10.1158/1078-0432.CCR-14-0442 25271081

[18] Yang R, Chang Q, Meng X, Gao N, Wang W. Prognostic Value of Systemic Immune-Inflammation Index in Cancer: A Meta-analysis. J Cancer. 2018;9(18):3295-302. doi: 10.7150/jca.25691. 10.7150/jca.25691 PMC6160683 30271489

[19] Xu M, Chen R, Liu L, Liu X, Hou J, Liao J, et al. Systemic Immune-inflammation Index and Incident Cardiovascular Diseases Among Middle-aged and Elderly Chinese Adults: The Dongfeng-Tongji Cohort Study. Atherosclerosis. 2021;323:20-29. doi: 10.1016/j.atherosclerosis.2021.02.012. 10.1016/j.atherosclerosis.2021.02.012 33773161

