# Supplementary material for: Relação entre o Índice Imune-inflamação Sistêmico e Circulação Colateral Coronariana em Pacientes com Oclusão Total Crônica
Source: Arq Bras Cardiol. 2022 Jun 6;119(1):69–75. [Article in Portuguese] doi: 10.36660/abc.20210414 (PMC9352117; doi:10.36660/abc.20210414)
Supplement: Translation

# Translation: Relationship between Systemic Immune-Inflammation Index and Coronary Collateral Circulation in Patients with Chronic Total Occlusion

**Authors:** Mehmet Koray Adali, Ipek Buber, Gursel Sen, Samet Yilmaz

**Affiliations:** 1 Turkey Pamukkale University - Faculty of Medicine, Departamento de Cardiologia, Denizli – Turkey

**Keywords:** Collateral Circulation, Coronary Occlusion, Coronary Vessels

## Abstract

**Background:**

Inflammation plays a key role in the initiation and progression of coronary artery disease (CAD). The systemic immune-inflammation index (SII) is a novel inflammatory parameter that has been shown to be associated with CAD.

**Objective:**

This study aimed to investigate the relationship between SII and coronary collateral circulation (CCC) in patients with stable CAD and chronic total occlusion (CTO).

**Methods:**

The patients were divided into two groups, with poor CCC and good CCC, according to the Rentrop Classification. Ninety-four patients had poor CCC, and 81 patients had good CCC. Inflammation parameters were calculated from the laboratory results. The statistical significance level applied was 0.05.

**Results:**

High SII level (OR: 1.003, 95% CI: 1.001-1.004, p<0,001), absence of CTO in RCA (OR: 0.204, 95% CI: 0.096-0.436, p<0,001) and low Gensini score (OR: 0.980, 95% CI: 0.962-0.998, p=0,028) were significantly associated with poor CCC. The cutoff value of SII was 679.96 for the highest predictive power of poor CCC, with a sensitivity of 74.5% and specificity of 43.2%. Mortality rates were similar between the two groups during a mean follow-up of 21.5±10.8 months (p=0.107).

**Conclusions:**

High SII level, the absence of CTO in the right coronary artery, and low Gensini score were significantly related to poor CCC. The rapid and cost-effective use of new inflammatory markers in clinical practice guides the prognosis of CAD.

## Introduction

Chronic total occlusion (CTO) is a type of coronary artery disease (CAD) characterized by complete or near-complete occlusion of the epicardial coronary arteries for at least three months and has worse clinical outcomes. CTO has an incidence ranging from 18% to 52% in the cohort obtained from the examination of coronary angiographies.^1^ Coronary collateral circulation (CCC) is an adaptive response that develops to maintain perfusion of myocardial tissue in patients with stenotic or occlusive coronary lesions. In a meta-analysis by Meier et al., it was reported that patients with good CCC had 36% less mortality than patients with poor CCC.^2^

The degree of coronary stenosis, presence of diabetes mellitus, exercise status, anginal attacks, mediators that affect angiogenesis such as vascular endothelial growth factor (VEGF), and the levels of inflammatory cells affect coronary collateral development.^2 - 6^ Because of the inflammatory processes that affect CAD on a large scale, inflammatory parameters obtained from routine tests such as complete blood count (CBC) and blood biochemistry are frequently used in a wide variety of clinical studies as predictors of both coronary collateral development and CAD severity.^7 - 9^

Systemic immune-inflammation index (SII), a novel inflammatory parameter, was found to be an independent predictor of cardiovascular events in CAD patients undergoing percutaneous coronary intervention (PCI).^10^ Although many inflammatory parameters have been studied in CAD patients with CTO, monocyte to high-density lipoprotein ratio (MHR) and SII have not been previously studied in the literature in this clinical situation. Therefore, we aimed to investigate the predictor value of SII on coronary collateral development in patients with stable CAD with CTO.

## Methods

### Study population and design

After the approval of the local ethics committee, 2576 coronary angiography procedure results were assessed between January 2018 and July 2020, obtained from the institute records. The flowchart of patient enrollment is seen in Figure 1 . One hundred and seventy-five stable CAD patients with CTO were included in the study and were grouped according to the Rentrop classification,^11^ in terms of coronary collateral development in CTO. The patients were divided into two groups, with poor CCC (Grades 0 and 1) and with good CCC (Grades 2 and 3). Ninety-four patients had poor CCC, and 81 patients had good CCC. Clinical and demographic characteristics, CAD risk factors, medications, laboratory results, electrocardiogram (ECG), and mortality recordings of the patients were obtained from the hospital database. SII, MHR, platelet to lymphocyte ratio (PLR), and neutrophil-lymphocyte ratio (NLR) were calculated from the CBC and biochemical parameters laboratory results. The SII value was calculated with the formula SII = (P × N) / L. In the formula, P, N, and L symbolize platelets, neutrophils, and lymphocytes, respectively. Hypertension was defined as the previous documentation of a systolic blood pressure of 140 mm Hg and/or a diastolic blood pressure of 90 mm Hg in at least two measurements or active use of any antihypertensive agent. Diabetes mellitus was defined as a fasting plasma glucose level > 126 mg/dL, a glucose level > 200 mg/dL, or a glycated hemoglobin level over 6.5% in any measurement, or the active use of an antidiabetic agent. Total cholesterol >200 mg/dL and triglyceride levels >150 mg/dL were considered as hyperlipidemia, or the active use of an antihyperlipidemic drug.

**Figure 1:** Flowchart of patient enrollment.

Patients with moderate to severe heart valve pathology, acute coronary syndrome in last three months, decompensated heart failure (NHYA class III or IV), chronic obstructive pulmonary disease, clinical signs of active infection, acute or chronic renal, hepatic insufficiency, and those with a history of malignancy, coronary artery bypass grafting (CABG) surgery, pulmonary embolism, chronic inflammatory or autoimmune diseases, and those undergoing renal-hepatic transplantation were excluded from the study.

This study complies with the principles outlined in the Declaration of Helsinki.

### Coronary collateral circulation assessment

Coronary angiography was indicated in patients with chest pain or those submitted to non-invasive tests that showed myocardial ischemia. The coronary angiography was performed by transfemoral or transradial access using the routine Judkins technique. CTO was defined as a total occlusion of a coronary artery with a distally TIMI 0 flow for at least 3 months. Patients who had at least one coronary artery with CTO were included in the study. CCC was evaluated by two cardiologists who were blinded to the study. CCC was graded using the scoring system developed by Cohen et al. (the Rentrop classification ).^11^ According to the classification system: Grade 0, no visible filling from any coronary collateral; Grade 1, filling of side branches of the artery to be dilated via collateral channels without visualization of the epicardial part; Grade 2, partial filling of the epicardial part via collateral channels; Grade 3, complete filling of the epicardial artery, being dilated via collateral channels.

### Statistical analysis

All data were analyzed using the SPSS 22.0 statistics package (SPSS Inc., Chicago, IL, USA). Continuous variables were reported as mean ± standard deviation, and categorical variables as absolute and relative frequencies. The Kolmogorov-Smirnov test was used to determine the normality of the data. The independent Student’s t-test was used to compare normally-distributed variables. Categorical variables were compared with the χ^2^test or Fisher’s exact test. A p-value < 0.05 was considered statistically significant. The effects of different variables on poor CCC were assessed by backward logistic regression analysis. The inclusion of covariates in the multivariate model was first determined by selecting those that exhibited 2-sided p < 0.10 in the unadjusted analyses. The inclusion of additional covariates was determined by performing a stepwise-backward selection process until all the other variables in the model exhibited p < 0.10. The receiver-operating characteristic (ROC) curve analysis was used to determine the best cutoff value of the SII level in predicting poor CCC.

## Results

In total, 175 stable CAD patients with CTO were enrolled in the study. The mean age of the patients was 68.2±10.9 and 80.6% of the patients were male. There were two groups; one that had 94 patients in the poor CCC (Rentrop Grade 0 or 1) and 81 patients in the good CCC (Rentrop Grade 2 or 3) groups. Age, gender, presence of hypertension, diabetes hyperlipidemia, family history of cardiovascular disease (CVD), prior MI, and medications were similar between the two groups. In all patients, the CTO location was higher in the right coronary artery (RCA) and statistically higher in the good CCC group. Multivessel disease (≥ 2 CAD) rate was slightly and Gensini score was significantly higher in the good CCC. Mortality rates were similar between the two groups, during a mean follow-up of 21.5±10.8 months. Baseline demographic, clinical characteristics, CAD risk factors, and previous medication of the patients are shown in Table 1 .

**Table 1:** Baseline demographic and clinical characteristics of the study population

Characteristics	All patients (n = 175)	Coronary collateral circulation		p-value

		Poor (n = 94)	Good (n = 81)	
Age (years), mean±SD	68.2±10.9	69.1±11.2	67.3±10.5	0.275
Male, n (%)	141 (80.6)	74 (78.7)	67 (82.7)	0.568
SBP, mm Hg	138.4±20.44	127.8±16	129.7±18.6	0.478
DBP, mm Hg	74.19±12.79	76.1±11.6	76.7±13	0.742
Current smoker, n (%)	36 (20.6)	18 (19.1)	18 (22.2)	0.708
Hypertension, n (%)	103 (58.9)	59 (62.8)	44 (54.3)	0.283
Diabetes mellitus, n (%)	69 (39.4)	38 (40.4)	31 (38.2)	0.877
Hyperlipidemia, n (%)	15 (8.6)	10 (10.6)	5 (6.1)	0.418
Family history of CVD, n (%)	15 (8.6)	10 (10.6)	5 (6.1)	0.418
Prior MI, n (%)	77 (44)	44 (46.8)	37 (45.7)	0.448
**Medication, n (%)**				
ASA	92 (52.6)	56 (59.6)	36 (44.4)	0.050
P2Y12 inhibitor	42 (24)	27 (28.7)	15 (18.5)	0.155
Statin	52 (29.7)	29 (30.8)	23 (28.4)	0.743
ACEI/ARB	71 (40.6)	40 (42.5)	31 (38.2)	0.644
Beta blocker	83 (47.4)	48 (51)	35 (43.2)	0.363
Calcium channel blocker	31 (17.7)	18 (19.1)	13 (16)	0.692
EF, %, mean±SD	47.1±12.2	46.1±12.2	48.3±12.2	0.224
Multivessel disease, n (%)	121 (69.1)	59 (62.8)	62 (76.5)	0.071
**CTO location, n (%)**				
LAD	46 (26.3)	30 (31.9)	16 (19.7)	0.085
Cx	25 (14.3)	16 (17)	9 (11.1)	0.287
RCA	96 (54.9)	38 (40.4)	58 (71.6)	<0.001
Other	22 (12.6)	13 (13.8)	9 (11.1)	0.652
Gensini score, mean±SD	58.6±23.2	55±18.6	62.8±27.1	0.025
Mortality, n (%)	41 (23.4)	27 (28.7)	14 (17.3)	0.107

*ACEI: Angiotensin-converting enzyme inhibitor; ARB: Angiotensin receptor blocker; ASA: Acetyl salicylic acid; CTO: Chronic total occlusion; CVD: Cardiovascular disease; Cx: Circumflex coronary artery; DBP: Diastolic blood pressure; EF: Ejection fraction; LAD: Left anterior descending coronary artery; MI: Myocardial infarction; RCA: Right coronary artery; SBP: Systolic blood pressure.*

The laboratory results and the inflammatory parameters of both groups are shown in Table 2 . Platelet levels, WBC and neutrophil counts were remarkably higher in the poor CCC group. Lymphocyte count was higher in the good CCC group. Hemoglobin, monocyte count, glomerular filtration rate, and cholesterol levels were similar between the two groups. Among the inflammatory parameters, C-reactive protein (CRP) and MHR showed no significant difference between groups, but NLR, PLR, and SII values were found to be statistically lower in the good CCC group.

**Table 2:** Laboratory results and inflammatory parameters of the patients

Characteristics	All patients (n = 175)	Coronary collateral circulation		p-value

		Poor (n = 94)	Good (n = 81)	
**Laboratory results, mean±SD**				
Hemoglobin, g/L	13.2±2	13±2.2	13.5±1.8	0.139
Platelet count, 10^3^/µL	253.8±60.4	267.5±65.2	237.9±50.1	0.001
WBC count, 10^3^/µL	9.4±2.8	10.1±3.1	8.7±2	0.001
Neutrophil count, 10^3^/µL	6.5±2.5	7.3±2.9	5.6±1.6	<0.001
Lymphocyte count, 10^3^/µL	2.1±0.9	1.9±0.85	2.2±0.88	0.028
Monocyte count, 10^3^/µL	0.62±0.25	0.62±0.26	0.62±0.25	0.251
Creatinine, mg/dL	1.04±0.28	1.04±0.27	1.04±0.29	0.895
GFR, mL/min	74.1±20.1	73.2±19.9	75.1±20.5	0.526
Total cholesterol, mg/dL	180.9±46.3	126±70.3	113±46.3	0.583
HDL-C, mg/dL	40.8±12.3	42±11.4	39.5±13.2	0.174
LDL-C, mg/dL	107.4±42.8	104.3±43.2	110.8±42.3	0.319
Triglycerides, mg/dL	171±112	167.9±99.8	174.6±125.2	0.694
CRP, mg/L	13.2±22	14.2±23.4	12.1±20.2	0.533
MHR	17.1±10.1	15.9±8	18.4±12	0.112
NLR	4.1±3.7	5.1±4.7	2.9±1.3	<0.001
PLR	152.5±108.7	179.5±136.7	121.2±46	<0.001
SII	1030.6±1008.9	1335.3±1275.4	679.9±295.3	<0.001

*CRP: C-reactive protein; GFR: Glomerular filtration rate; HDL-C: High density lipoprotein cholesterol; LDL-C: Low density lipoprotein cholesterol; MHR: monocyte to high-density lipoprotein ratio; NLR: neutrophil to lymphocyte ratio; PLR: platelet to lymphocyte ratio; SII: systemic immune-inflammation index; WBC: total white blood cells.*

The multivariate Backward-Regression analysis of risk factors for poor CCC was performed. The model included age, gender, hypertension, diabetes, hyperlipidemia, current smoking, prior MI, multivessel disease, heart rate, ejection fraction, acetylsalicylic-acid use, statin use, presence of CTO in the RCA, collateral state, Gensini score, NLR, PLR, and SII. The analysis showed that the absence of CTO in RCA and low Gensini score were related to poor CCC. In addition, a high SII level was significantly associated with poor CCC ( Table 3 ).

**Table 3:** Multivariate Backward-Regression analysis of risk factors for poor CCC

Variables *,**,***	OR (95% CI)	p value
Hyperlipidemia	0.313 (0.091-1.071)	0.064
RCA CTO	0.204 (0.096-0.436)	<0.001
Gensini score	0.980 (0.962-0.998)	0.028
ASA use	0.526 (0.249-1.111)	0.092
SII	1.003 (1.001-1.004)	<0.001

ASA: Acetyl salicylic acid; CCC: Coronary collateral circulation; CI: Confidence interval; RCA: Right coronary artery; OR: Odds ratio; SII: systemic immune-inflammation index. *Nagelkerke R square: 0.432. **The model included age, gender, hypertension, diabetes, hyperlipidemia, current smoking status, prior MI, multivessel disease, heart rate, ejection fraction, acetylsalicylic-acid usage, statin use, presence of chronic total obstruction in the right coronary artery, collateral grade, Gensini score, NLR, PLR, and SII. ***Selection of the covariates for the multivariate models is explained in the Methods section. Unless otherwise indicated, odds ratio is interpreted as the presence (vs. absence) of each categorical variable or an increase of one (1) unit of each continuous variable.

We assessed the predictor value of the SII for poor CCC in a ROC curve analysis. When the cutoff value of the SII was set at 679.96, the predictive power of poor CCC was the highest, with a sensitivity of 74.5% and specificity of 43.2% (AUC: 0.732; 95% CI, 0.659–0.804, p<0.001) ( Figure 2 ).

**Figure 2:** ROC curves of patients with poor CCC predicted by SII.

AUC: Area under curve; CCC: coronary collateral circulation; ROC: Receiver-Operating characteristics; SII: Systemic imune-inflammation index

## Discussion

To the best of our knowledge, this is the first study that evaluates the relationship between SII and CCC in patients with stable CAD and CTO. In the current study, we found that a high SII, the absence of CTO in RCA, and low Gensini score were related to poor CCC.

Coronary collateral vessels are an adaptive mechanism that is activated by chronic or recurrent myocardial ischemic events; they progress gradually, and protect from myocardial ischemia and its associated complications.^2 , 12^ Hypoxia, increased redox potential or shear stress, and some genomic expressions cause endothelial cell activation and initiation of the inflammatory cascades.^13^ Because of the central role of the inflammation on the initiation and progression of CAD, various studies have been carried out to identify the effect of inflammatory processes on CCC. High CRP, NLR, PLR, CRP to albumin ratio (CAR), and fibrinogen to albumin ratio (FAR) have been used for this purpose.^7 , 8 , 14 - 16^

Acar et al. found that PLR was a predictor of poor collateral flow in patients with stable angina pectoris and CTO.^7^ In another study, NLR was found to be associated with reduced coronary collateral flow in CAD with CTO.^8^ We also found the PLR and NLR levels were high in the poor CCC group (p <0.001), but this significance was not found in the regression analysis.

Increased MHR level has been identified as a predictor of the high SYNTAX score in stable CAD patients.^9^ In the current study, we also aimed to investigate the effect of this inflammatory parameter on CCC development, but there was no significant difference in terms of MHR.

SII has been developed from inflammatory cells including platelet, neutrophil, and lymphocyte counts. Firstly, it has been associated with poor prognosis in many types of cancer.^17 , 18^ Using The Dongfeng-Tongji cohort, Xu et al. have found that SII was associated with thrombocytosis, inflammation, and the development of cerebrovascular disease in 13,929 middle-aged and older adults without CVD and cancer, over a mean follow-up of 8.28 years.^19^ Yang et al. have demonstrated that high SII level is independently associated with increased risk of cardiovascular death, nonfatal MI, nonfatal stroke, and admission for heart failure in 5206 CAD patients who underwent PCI.^10^ In this study, an optimal SII cutoff point (≥694.3) was identified for major adverse cardiovascular events (MACE) in the CAD cohort. Similarly, in our study, we found an optimal SII cutoff point of 679.96 for the best prediction of poor CCC, with a sensitivity of 74.5% and a specificity of 43.2%.

The effect of the CCC on mortality is debatable. In a meta-analysis that included over 3000 patients, Allahwala et al. have indicated that robust CCC is not associated with lower rates of acute myocardial infarction or all-cause mortality but increases the chance of PCI success.^1^ On the other hand, Meier et al. demonstrated that high collateralization had a protective effect and a 36% decreased mortality risk compared with patients with low collateralization.^2^ However, in our study, there was no significant difference in mortality rates during 21.5±10.8 months of follow-up.

This study has some limitations. First, there was quite a small number of patients and the study was a cross-sectional, single-center one, with a retrospective design. Hence, the selected sample population may not reflect the whole cohort, and thus further studies are warranted. Second, all measurements and laboratory parameters were evaluated only once during follow-up. Finally, specific gene expressions, inflammatory parameters such as VEGF and TNF-α were not measured, so these measurements could be supportive in demonstrating the association of poor CCC with SII.

## Conclusion

In this study, we found that a high SII, the absence of CTO in RCA, and low Gensini score were significantly related to poor CCC. It is important to quickly determine the inflammation status from the blood laboratory results and to determine the poor CCC and high-risk patients that result in high mortality in CAD patients. SII is an inflammatory parameter, which is easy to calculate from CBC and may be very useful to identify high-risk patients with poor CCC.
